# Upper eyelid blepharoplasty following hyaluronic acid injection with improved facial aesthetics and eye symptoms: a case report

**DOI:** 10.1186/s13256-020-02641-0

**Published:** 2021-04-29

**Authors:** Luca Maione, Valeriano Vinci, Domenico Costanzo, Andrea Battistini, Andrea Lisa, Alessandra Di Maria

**Affiliations:** 1 Plastic Surgery Unit, Humanitas Clinical and Research Center – IRCCS, Via Manzoni 56, 20089 Rozzano, Milan, Italy; 2 Department of Medical Biotechnology and Translational Medicine BIOMETRA, Reconstructive and Aesthetic Plastic Surgery School, University of Milan, Rozzano, Milan, Italy; 3 Plastic Surgery Unit, Clinica San Carlo, Via Ospedale 21, 20037 Paderno Dugnano, Milan, Italy; 4 Department of Biomedical Sciences, Humanitas University, Via Rita Levi Montalcini 4, 20090 Pieve Emanuele, Milan, Italy; 5 Humanitas Clinical and Research Center-IRCCS, Via Manzoni 56, 20089 Rozzano, Milan, Italy; 6 Humanitas University (Hunimed), Rozzano, Milan, Italy; 7 Ophthalmology Unit, Humanitas Research Hospital, Rozzano, Milan, Italy

**Keywords:** Dermatochalasis, Blepharoplasty, Hyaluronic acid filler, Blepharoplasty outcomes evaluation, case report

## Abstract

**Background:**

Dermatochalasis of the upper eyelids (blepharochalasis) is a typical age-related change in the upper third of the face and a major concern for facial aesthetics. Nowadays both surgical and nonsurgical interventions are available for patients complaining of upper eyelid dermatochalasis. Although nonsurgical treatments are often easier to perform, if they are not performed correctly, complications may ensue and worsen the condition.

**Case presentation:**

We describe the case of a Caucasian patient presenting with bilateral upper eyelid dermatochalasis, previously treated with multiple injections of hyaluronic acid filler. Following these procedures, the patient reported nonspecific eye symptoms (such as a sense of heaviness and asthenopia) and cosmetic concerns. We decided to perform an upper eyelid blepharoplasty. During the procedure we found a ribbon of hard, fibrous material, which was carefully removed. The patient reported resolution of functional eye symptoms owing to the reduction of upper lid heaviness, which also resulted in subjective improvement of the visual field. Patient satisfaction was assessed preoperatively and 3 months postoperatively using the Blepharoplasty Outcomes Evaluation (BOE), which showed an overall satisfaction rate of 95.8 %.

**Conclusions:**

Blepharoplasty not only treated the patient’s blepharochalasis but also allowed us to correct the previous nonsurgical intervention by removing the excessive amount of injected hyaluronic acid. Both aesthetic and functional results were successfully achieved.

## Introduction

With advancing age, eyelid tissues may undergo dramatic morphological changes which involve elastolysis and collagen rearrangement and result in excess eyelid skin—a condition known as dermatochalasis.

Upper eyelid dermatochalasis may lead to aesthetic, functional and psychological issues and should be addressed by surgeons with specific expertise in ophthalmoplasty. Nowadays both surgical and nonsurgical interventions are available for patients complaining of upper eyelid dermatochalasis.

- Medical interventions include, among others, volume augmentation of the brow with hyaluronic acid (HA) fillers. They not only enable correction of age-related eyelid hollowing, but are also useful for post-blepharoplasty volume loss. However, effects are temporary and, if not performed correctly, complications may ensue and worsen the condition.
- Surgical interventions include upper eyelid blepharoplasty (exeresis of the excess musculocutaneous tissue), which can be both a definitive functional and cosmetic procedure [1].

In the present paper we describe the case of a patient presenting with dermatochalasis, who had been treated with multiple injections of HA in the upper eyelid, in which eyelid surgery resulted in improvement of both facial aesthetics and eye symptoms.

## Case presentation

The patient was a 75-year-old Caucasian woman who presented to our clinic in 2019 complaining of dermatochalasis of the upper eyelids (blepharochalasis) and asking for upper eyelid rejuvenation. Her past medical history was otherwise unremarkable. In the past 2 years she had been injected several times with an HA filler on her upper eyelids at a private clinic (she received three injections, once every 4 months), but the treatment worsened her clinical presentation. Indeed, an excessive amount of HA was injected (the patient reported more than 1.5 mL for each eyelid), and instead of improving the shape and contour, the filler caused increased weight of the superior eyelid, resulting in worsening of her symptoms.

**Fig. 1:**
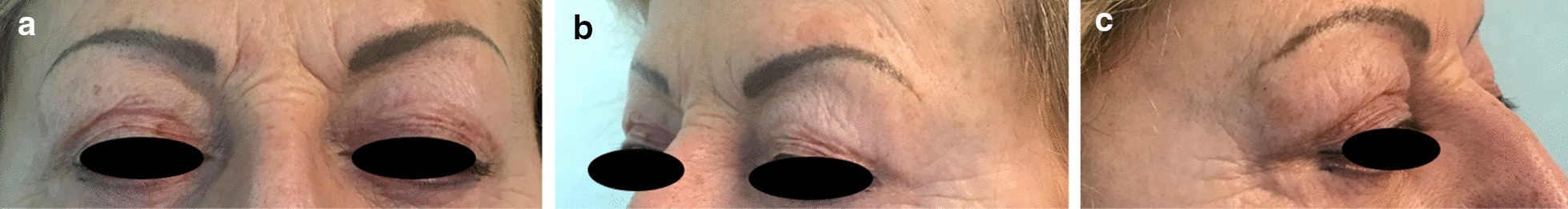
**a**–**c** Preoperative (frontal and lateral) view

She reported nonspecific eye symptoms, such as a sense of heaviness and asthenopia, and cosmetic concerns, as it gave the patient a tired and dull look to the face, compared to the situation before HA injection (Fig. 1).

After a thorough assessment of the patient, we decided that further medical treatments were not ideal in this situation; for this reason, we agreed that a bilateral upper eyelid blepharoplasty was the best choice.

Upon preoperative marking and injection of mepivacaine 20 mg/mL with adrenaline, we performed exeresis of the excess musculocutaneous tissue of the upper eyelids, appropriate hemostasis, opening of the superior orbitopalpebral fascia, removal of excess adipose tissue hernias and suturing of the skin flaps.

**Fig. 2:**
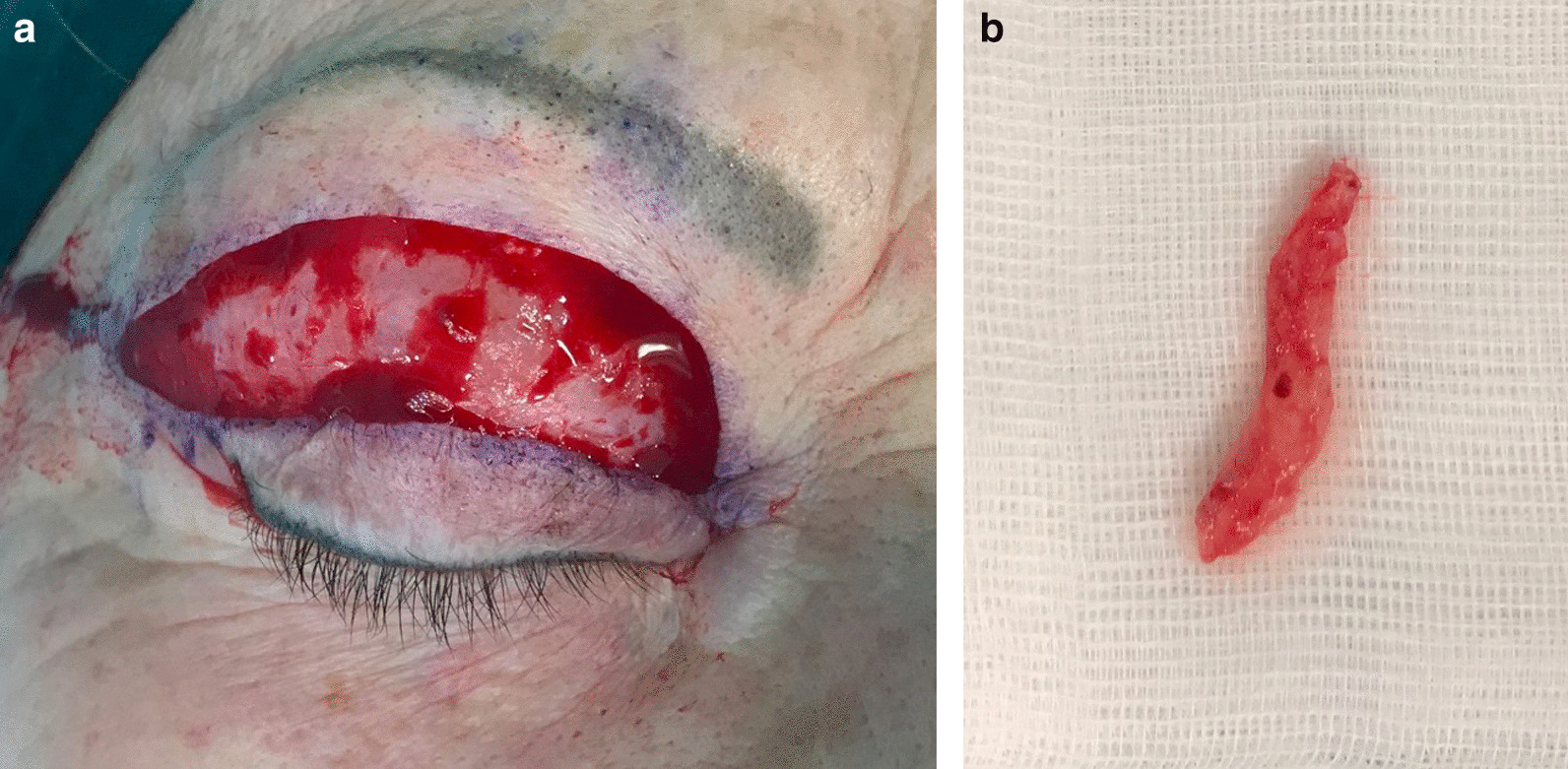
**a** Intraoperative view and **b** the ribbon of hard, fibrous material consistent with HA filler previously injected

During the procedure we found a ribbon of hard, fibrous material, which was carefully removed (Fig. 2).

Histopathological examination showed the presence of irregular, amorphous, light grayish to bluish material that separated from dispersed collagen bundles with sparse inflammatory cell infiltration in the lower dermis and subcutaneous areolar tissue. The amorphous material stained blue with Alcian blue, pH 2.5. These findings were considered to be consistent with HA.

**Fig. 3:**
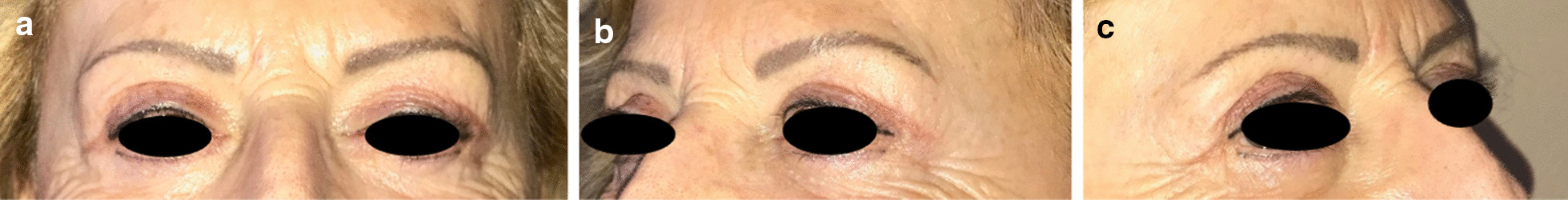
**a**–**c** Postoperative (frontal and lateral) view

The patient had a good recovery, and sutures were removed 1 week postoperatively. Superficial ecchymosis resolved in 10 days; no postoperative complications including superficial hematoma, wound dehiscence, scar abnormalities or upper eyelid overcorrection were observed (Fig. 3).

The aesthetic result obtained includes sharp and precise supratarsal crease with pretarsal show; appropriate lid position, with the upper lid extending down 2 mm below the upper limbus and the lower eyelid resting at the inferior limbus; and smooth lid-cheek junction.

The patient reported resolution of functional eye symptoms owing to the reduction of upper lid heaviness, which also resulted in subjective improvement of visual acuity.

**Fig. 4:**
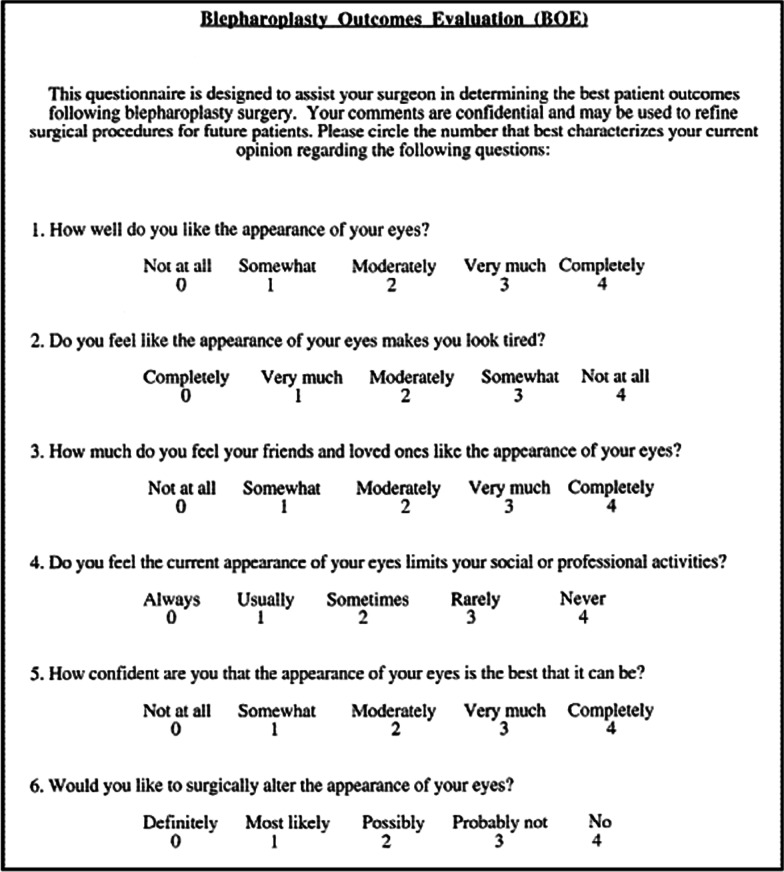
Blepharoplasty Outcomes Evaluation (BOE) questionnaire

Patient satisfaction was assessed pre- and postoperatively (3 months from surgery) using the Blepharoplasty Outcomes Evaluation (BOE), a six-item free questionnaire evaluating appearance, function and social acceptance. Each of the six items is scored on a scale of 0–4, with 0 representing the most negative response and 4 the most positive. Dividing the total score for each instrument by 24 and multiplying by 100 yields the scaled instrument score. This range is 0–100, with 0 representing the lowest patient satisfaction and 100 representing the highest patient satisfaction [2] (Fig. 4).

The overall satisfaction reported by the patient was 25% preoperatively, while her level of satisfaction 3 months after the procedure was 95.8 %.

## Discussion

From superficial to deep, the upper eyelid consists of skin and subcutaneous areolar tissue, striated muscle, submuscular areolar tissue, and the tarsi and the orbital septum. With aging, the upper eyelid undergoes dramatic morphological changes. Excess upper eyelid skin (dermatochalasis) is one of the manifestations of periorbital aging that, apart from creating an undesirable appearance, may also impair the function of the eye (e.g., lateral hooding with superolateral visual field obstruction).

Dermatochalasis is a consequence of time-related elastolysis and collagen rearrangement. [3] This causes a notable loss of ground substance and thus thinning of the dermis. The combination of these changes makes the skin less elastic. The cumulative effects of gravity on this less elastic skin and decreased subcutaneous tissue evolve into dermatochalasis. Subclinical inflammation with elastolysis and secondary lymphostasis may contribute to dermatochalasis.

Histological examination of dermatochalasis specimens shows an increased number and dilation of lymphatic vessels in conjunction with widely spaced collagen bundles and a reduction in elastic fibers. [4, 5]

It is possible to address the aforementioned changes in the upper eyelids with medical and/or surgical interventions.

### Soft tissue fillers

Different types of dermal fillers are currently used for nonsurgical aesthetic procedures to correct soft tissue and osseous volume loss in the periorbital region, with hyaluronic acid gel (HAG) fillers being the most commonly preferred agent. The particular characteristics of the filler should be carefully considered in this region [6–8]

Complications related to HAG fillers can be categorized as early (less than 14 days, such as erythema, edema, bruising, lumps, infection, arterial embolism), late (from 14 days to 1 year, such as foreign body granuloma reaction) and delayed (more than 1 year, such as biofilms) [9–17].

In our practice, we have performed injections in the supraperiosteal plane (with large-particle HA fillers) for augmentation of the brow to correct atrophy of the retro-orbicular fat pad; if not used correctly (or overused), such fillers may lead to brow ptosis and worsen dermatochalasis.

When dealing with large HA particles, it is important to avoid superficial injections, which can lead to contour irregularities and a bluish discoloration secondary to the Tyndall effect [18, 19].

However, the main disadvantage of HAG fillers is their temporary nature.

### Upper eyelid blepharoplasty

Before starting the procedure, preoperative markings are drawn with the patient sitting upright and looking directly ahead (neutral gaze). The lower limit of excision is represented by the upper eyelid crease. The superior extent of skin excision should be at least 10 mm from the inferior border brow; the lateral one is defined by an oblique line from the lateral canthus to the lateral end of the brow. Upon infiltration with a local anesthetic, excision of the marked skin is performed. The medial fat compartment can be accessed by incising the orbital septum, while the retro-orbicularis oculi fat can be resected by dissecting beneath the lateral orbicularis oculi. At least 20 mm of vertical lid height should be preserved to facilitate normal eye closure [20]. Closure of the resected skin margins is achieved with either absorbable or permanent suture.

In the present case we observed incorrect use of HA filler for the treatment of upper blepharochalasis, which resulted in a worsening of the original clinical presentation. Based on our experience, we treated dermatochalasis with blepharoplasty [21]. Not only were we able to address the aesthetic concern in removing the excess skin, but surgery also revealed the presence of foreign material: a ribbon of hard, fibrous material consistent with HA resulting from previous inappropriate filler injections. The patient was satisfied with the treatment from both an aesthetic and functional point of view: she reported resolution of eye symptoms owing to the reduction of upper lid heaviness, which also resulted in subjective improvement of the visual field.

## Conclusions

HA fillers are an excellent tool for correcting volume loss of the upper eyelids, improving the contour and correcting asymmetries; additionally, they are extremely useful for optimizing surgical outcomes. However, if not used correctly, complications may ensue and worsen the condition. In these cases, a surgical approach can achieve resolution. In the present case, blepharoplasty not only treated the patient’s blepharochalasis but also allowed us to correct the previous nonsurgical intervention by removing the excessive amount of injected HA. Both aesthetic and functional results were successfully achieved.

## Data Availability

Data sharing is not applicable to this article as no datasets were generated or analyzed during the current study.
